# Solving the RNA design problem with reinforcement learning

**DOI:** 10.1371/journal.pcbi.1006176

**Published:** 2018-06-21

**Authors:** Peter Eastman, Jade Shi, Bharath Ramsundar, Vijay S. Pande

**Affiliations:** 1 Department of Bioengineering, Stanford University, Stanford, CA, United States of America; 2 Department of Chemistry, Stanford University, Stanford, CA, United States of America; 3 Department of Computer Science, Stanford University, Stanford, CA, United States of America; University of Missouri, UNITED STATES

## Abstract

We use reinforcement learning to train an agent for computational RNA design: given a target secondary structure, design a sequence that folds to that structure *in silico*. Our agent uses a novel graph convolutional architecture allowing a single model to be applied to arbitrary target structures of any length. After training it on randomly generated targets, we test it on the Eterna100 benchmark and find it outperforms all previous algorithms. Analysis of its solutions shows it has successfully learned some advanced strategies identified by players of the game Eterna, allowing it to solve some very difficult structures. On the other hand, it has failed to learn other strategies, possibly because they were not required for the targets in the training set. This suggests the possibility that future improvements to the training protocol may yield further gains in performance.

## Author summary

Designing RNA sequences that fold to desired structures is an important problem in bioengineering. We have applied recent advances in machine learning to address this problem. The computer learns without any human input, using only trial and error to figure out how to design RNA. It quickly discovers powerful strategies that let it solve many difficult design problems. When tested on a challenging benchmark, it outperforms all previous algorithms. We analyze its solutions and identify some of the strategies it has learned, as well as other important strategies it has failed to learn. This suggests possible approaches to further improving its performance. This work reflects a paradigm shift taking place in computer science, which has the potential to transform computational biology. Instead of relying on experts to design algorithms by hand, computers can use artificial intelligence to learn their own algorithms directly. The resulting methods often work better than the ones designed by humans.

## Introduction

### The RNA design problem

Re-engineering and *de novo* design of RNA molecules to perform novel biological functions have been major focuses of modern bioengineering research [1–5]. The function of an RNA molecule is determined by the structure into which it folds, which is in turn determined by the sequence of nucleotides that comprise it. Designing RNA molecules to perform specific functions therefore requires solving the inverse folding problem for RNA: given a target structure, design a sequence that folds into that structure. Here, we are specifically interested in solving the *computational* RNA design problem: given a target structure, design a sequence that folds into the target as judged by an *in silico* structure predictor. Current state-of-the-art structure prediction software such as ViennaRNA [6] can predict the experimental minimum free energy structure with high accuracy, and has been a key step in designing RNA molecules for several real-life biological applications [7–10]. Therefore, solving the computational RNA design problem will likely provide valuable insights into solving real-life RNA design problems.

Significant progress has been made in developing machine-based algorithms for computational RNA design. One of the initial algorithms, RNAInverse [11], uses a simple adaptive walk in which random single or pair mutations are performed on a sequence, and a mutation is accepted if it improves the structural similarity between the current and the target structure. A subsequent algorithm RNA-SSD [12] first performs hierarchical decomposition of the structure into substructures to reduce the size of the search space before performing adaptive walk. INFO-RNA [13] first generates an initial guess of the sequence using dynamic programming to estimate the minimum energy sequence for a target structure, and then performs a stochastic search using simulated annealing. DSS-Opt [14] and NUPACK [15] both attempt to optimize more complex objective functions that explicitly punish incorrectly-pairing bases. DSS-Opt employs a gradient-based approach on the full sequence to optimize an objective function that includes both a free energy component and a “negative design term” to punish incorrectly paired bases. On the other hand, NUPACK first performs hierarchical decomposition on the target, and then for each substructure, computes a thermodynamic ensemble of structures given the current sequence. It then perturbs the sequence to optimize the “ensemble defect”, which is the average number of incorrectly paired bases across all structures in the ensemble, weighted by their thermodynamic populations. Finally, MODENA [16] generates an ensemble of initial guesses using a genetic algorithm, and then performs stochastic search using either crossover moves, in which pieces of two candidate solutions at the same position are swapped with each other, or single-point mutations. These solutions are then judged using an objective function with components for both energetic stability and target structure similarity.

Eterna [17] takes a very different approach to the problem. It is an online open laboratory that presents the inverse folding problem as a game, and asks players to create sequences that fold to specific structures. Some of the top players develop exceptional skill at this task, and can successfully find solutions for structures that none of the algorithms described above can solve.

The Eterna100 benchmark [18] is a collection of 100 target structures ("puzzles") created by players of the Eterna game. They were chosen to cover a wide range of difficulty levels, and to exhibit a variety of challenging structural elements. They are ordered inversely with respect to the overall number of correctly proposed solutions by Eterna players, with the second half being significantly more difficult to solve than the first half. A test of the above algorithms on the Eterna100 benchmark resulted in several of the algorithms performing poorly, with RNAInverse and RNA-SSD predicting valid sequences for only 28/100 and 27/100 structures respectively. Even the best-performing algorithm, MODENA, could only succeed on 54/100 structures.

A common weakness of several existing computational algorithms, including RNAInverse, RNA-SSD, INFO-RNA, NUPACK, and MODENA, is that they rely at least partially on purely stochastic search: random mutations are made to a sequence and beneficial mutations (e.g. that improve structural similarity to the target or reduce the structure’s energy) are accepted. This procedure successfully predicts sequences for short nucleotide chains and simple RNA structures due to the small space of possible mutations. However, this method fails for longer chains or more complex structures in which sampling the entire space of possible mutations is prohibitively expensive. One way to reduce the size of the search space is to first decompose the full structure into substructures and independently optimize each substructure, as was done in RNA-SSD. However, for target structures in which accurate determination of the sequence of one part of the structure requires global knowledge about the entire structure, hierarchical decomposition is not feasible. For example, the "Mutated Chicken Feet" structure from the Eterna100 contains three symmetric branches in which each branch must be assigned a sequence that is different from the other two, and thus cannot be solved by independently considering each branch. Alternatively, target structures that possess easily accessible off-pathway local minima to obstruct the search, or lack on-pathway local minima to positively guide the search, present additional challenges for these algorithms.

One possible means to address the weaknesses of purely stochastic search is to incorporate prior information into the model. Most commonly this is done by performing free energy calculations to find an energetically favorable candidate sequence for a given target that is closer to a valid sequence, thereby reducing the number of search steps. Algorithms such as INFO-RNA use this strategy, in which it first performs base-by-base free energy calculations over the target structure to generate the minimum energy sequence for the target. This sequence is then used as a starting point for the subsequent stochastic search. This method results in significantly increased performance on the Eterna100, with INFO-RNA succeeding on 50/100 structures as opposed to RNAInverse’s 28/100. Nevertheless, this strategy proves infeasible in many cases because the sequence that minimizes the energy for a target structure is not guaranteed to fold into that structure, or even a structure close to it. For many of the more complex puzzles in the second half of the Eterna100, the sequence which minimizes the energy of the target possesses a global energy minimum that is structurally very different from the target, making it very difficult to refine this solution into a correct one through stochastic search. Algorithms such as DSS-Opt and NUPACK attempt to mitigate this issue by using more complex objective functions that punish incorrectly paired bases. This allows for some level of control in avoiding sequences that fold into structures very different from the target structure, leading to respectable performance on the Eterna100, with DSS-Opt and NUPACK succeeding on 47/100 and 48/100 structures respectively. Most notably, both DSS-Opt and NUPACK manage to succeed on some structures unsolvable by INFO-RNA, such as "Misfolded Aptamer 6", demonstrating the advantages of utilizing a more complex objective function for certain structures. Nevertheless, the performance of all the algorithms substantially decreases as puzzle complexity increases, as all perform very poorly on the second half of the Eterna100, solving at maximum only about 25% of the puzzles.

### Reinforcement learning

In the last decade, machine learning has had remarkable success at solving a variety of challenging computational problems including computer vision [19], speech recognition [20], machine translation [21], and others. Instead of designing an algorithm by hand, one constructs a very flexible mathematical model (usually a neural network), then optimizes the parameters of the model until it produces the desired output for a set of training data.

Reinforcement learning (RL) is a branch of machine learning that deals with problems where an agent performs a series of actions to reach a goal. In the past, RL has proven extremely effective at training agents to perform a variety of difficult tasks, from video game playing [22] to robotic arm control [23]. Most recently, AlphaGo Zero achieved superhuman performance in the game of Go by learning the game purely through self-play [24]. Given these remarkable results, we reasoned that RL might be able to train a competent agent for RNA design. Similar to how an agent for Go can learn the optimal move to make given one of many unique board positions, we hypothesized that an RNA design agent could also learn the optimal design choice given one of many unique target RNA structures.

Reinforcement learning describes a problem in terms of an agent (e.g. a player of the game Eterna) interacting with an environment (e.g. an RNA molecule). At each step, the agent observes the current state of the environment (the current RNA sequence), and selects an action to perform (a change to the sequence). The action is selected by following a policy represented by a neural network. Whenever the agent makes progress toward its goal, it receives a reward. The policy network is trained by having the agent perform the task over and over, adjusting the network parameters to maximize the expected future rewards.

Remarkably, this simple process can sometimes produce algorithms that outperform the very best algorithms crafted by expert programmers. In this work, we apply it to the RNA inverse folding problem, training a policy network that outperforms all previous algorithms on the Eterna100 benchmark.

## Methods

### Overview

We formulate the inverse folding problem as a reinforcement learning problem. The current state corresponds to a candidate sequence. The agent takes actions that modify the type of a single base, or in some cases two paired bases. We train a policy network to select actions that eventually lead to a sequence with the desired secondary structure. When the target structure is achieved (that is, when the agent finds any sequence that is predicted to fold to the target structure), the agent receives a fixed positive reward. All other actions receive a reward of 0.

The input to the policy network is an N×4 tensor, where N is the number of bases, giving the current sequence in one-hot encoding. The network's output is an N×4 tensor containing action probabilities. Each element is the probability of changing one of the N bases to one of the four possible types. At each step, an action is randomly chosen based on the probabilities generated by the network. Note that 25% of all actions simply say that a base should have the same type it already has. To avoid wasting time on unproductive actions, the probabilities of these actions are forced to be 0.

If an action modifies a base that is supposed to be paired in the target structure, the type of its desired partner is checked and, if necessary, modified as well to ensure the two bases are capable of forming a pair. If their types are not one of GC, AU, or GU, the target partner is modified so they can form a GC or AU pair.

### Network architecture

Our goal is to train a single model that can be applied to RNA sequences of any length. This requires that the set of parameters defining the model must be independent of sequence length. We achieve this by building the model entirely out of convolutional layers.

More precisely, each layer takes as input a tensor of shape N×C_in_ and produces one of shape N×C_out_,where C_in_ and C_out_ are the numbers of channels per base. At the network's input (the current sequence) and output (action probabilities), the number of channels is 4. At all other points in the network, it is set to 80. The network is built up out of the following types of layers.

*Single base convolution (conv1)*: The output is computed independently for every base. Each output channel is a linear combination of the input channels for that same base.

*Seven base convolution (conv7)*: The output for each base is a linear combination of the inputs for seven bases, followed by a ReLU activation. The seven bases include:

- The five consecutive bases centered at that base
- The base with which that base should be paired in the target structure
- The base with which that base is actually paired in the structure formed by the current sequence

For example, suppose that base 7 is supposed to be paired with base 18 in the target structure, but is actually paired with base 15 in the structure to which the current sequence folds. The output for base 7 would be calculated from the inputs for bases 5, 6, 7, 8, 9, 18, and 15.

It is possible for some of these bases not to exist, such as if the target base is at the end of the chain, or if it is not paired in the target structure. In that case, the corresponding inputs are set to 0.

The *conv7* layer can be viewed as a type of graph convolution, although it is unusual in that the graph structure may change at every step, as it explores sequences that fold to different structures. Also note that the connections formed within *conv7* layers are the only way in which the network receives information about the target structure.

Instead of using a simple convolutional network, we combine multiple layers to form residual blocks. The output of each block is computed as

**y** = **x** + conv1(conv7(**x**))

Networks composed of residual blocks have been shown to be easier to train and to produce better results than simple convolutional networks [25].

**Fig 1:**
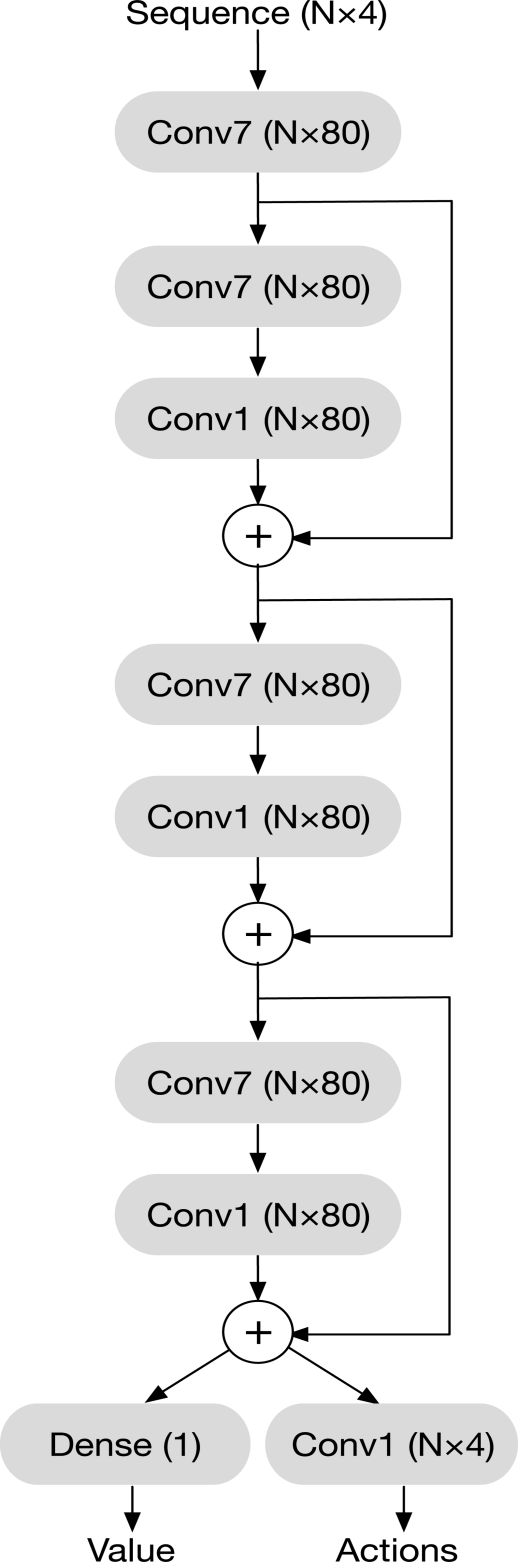
The architecture of the policy network. The shape of each layer's output is given in parentheses, where N is the number of bases in the sequence. "Conv1" and "Conv7" indicate single base convolution and seven base convolution layers, as described in the text. "Dense" indicates a dense (fully connected) layer, where every output depends on every input.

The full network is shown in Fig 1. It consists of a single *conv7* layer that increases the number of channels from 4 to 80, followed by three residual blocks. A final *conv1* layer with softmax activation computes the action probabilities. In addition, a dense (fully connected) layer computes an estimate of the state value function, which is used by the training algorithm [26]. It outputs an estimate of the total future reward starting from a given state. The number of parameters in the dense layer depends on the length of the sequence, which means it is necessarily specific to a particular sequence length. It is only used during training, however. Therefore, all training sequences must have the same length, but once training is complete, the policy network can be applied to sequences of any length.

The numbers, types, and widths of the layers in the network were chosen by experimentation, although no exhaustive hyperparameter search was performed. Our goal was to find the smallest network such that further increases in size had little benefit. For example, using 80 output channels was found to give significantly better results than 64, but 100 output channels worked no better than 80. A more rigorous hyperparameter search might improve our results somewhat, but would probably not dramatically change the model's performance.

### Training procedure

We require a set of target structures to use for training. We created them by randomly generating 100,000 RNA sequences of length 32, then computing the structure each one folds to. This yielded a total of 46,188 unique structures, none of which appears in the Eterna100 benchmark. The majority of them (34,264) occurred only a single time among the 100,000 sequences. Another 6158 structures occurred twice, and 5766 structures occurred three or more times. The number of times a structure occurred gives an indication of its difficulty: if many sequences fold to a structure, it will be much easier to find one than if only few sequences do. Only the structures were used in training, not the sequences. Any sequence that folded to a target structure was accepted as a valid solution.

The network was trained using the Asynchronous Advantage Actor-Critic (A3C) algorithm [26]. A different target structure was randomly chosen for every episode. Training was run for a total of 1.5 million steps. The learning rate was initially set to 10^−5^, then decreased by a factor of 0.8 after every 100,000 steps.

The first half million steps used only the easier structures for training (those which occurred three or more times). For the final 1 million steps, all structures were used except for 500 of the most difficult ones (ones that occurred only a single time), which were set aside for use as a validation set. Every 100,000 steps, the current network was used to solve all of the validation structures and the total number of steps requires was recorded. If this was less than the previous best validation score, the network parameters were saved as the new best network.

The model and training procedure were implemented using DeepChem 1.3.1 [27] and Tensorflow 1.3 [28]. ViennaRNA 2.3.5 [6] was used to compute the folded structures for sequences.

### Testing procedure

The trained model was tested on the Eterna100 benchmark by following procedures that, as closely as possible, match those used in [18]. For each puzzle, a random sequence was initially chosen, then modified by performing actions chosen by the policy network until a solution was reached. 24 hours were allowed for each attempt, and up to five attempts were made for each puzzle. Each attempt ran on a single Intel Xeon E5-2680 v2 CPU.

## Results and discussion

In total, our method successfully solved 60 of the 100 puzzles. For comparison, of the six algorithms tested in [18], the best benchmark score was for MODENA which solved 54 puzzles. Our method thus outperforms all previous algorithms. Full results are shown in Table 1.

**Table 1:** Puzzles in the Eterna100 benchmark. The ones our method succeeding in solving are marked with an X.

1. Simple Hairpin	X	51. medallion	X
2. Arabidopsis Thaliana 6 RNA—Difficulty Level 0	X	52. [RNA] Repetitious Sequences 8/10	
3. Prion Pseudoknot—Difficulty Level 0	X	53. Documenting repetitious behavior	
4. Human Integrated Adenovirus—Difficulty Level 0	X	54. 7 multiloop	X
5. The Gammaretrovirus Signal—Difficulty Level 0	X	55. Kyurem 7	X
6. Saccharomyces Cerevisiae—Difficulty Level 0	X	56. JF1	X
7. Fractal 2	X	57. multilooping fun	
8. G-C Placement	X	58. Multiloop…	X
9. The Sun	X	59. hard Y	X
10. Frog Foot	X	60. Mat—Elements & Sections	
11. InfoRNA test 16	X	61. Chicken feet	
12. Mat—Martian 2	X	62. Bug 18	
13. square	X	63. Fractal star x5	X
14. Six legd turtle 2	X	64. Crop circle 2	
15. Small and Easy 6	X	65. Branching Loop	X
16. Fractile	X	66. Bug 38	
17. Six legd Turtle	X	67. Simple Single Bond	
18. snoRNA SNORD64	X	68. Taraxacum officinale	
19. Chalk Outline	X	69. Headless Bug on Windshield	
20. InfoRNA bulge test 9	X	70. Pokeball	
21. Tilted Russian Cross	X	71. Variation of a crop circle	
22. This is ACTUALLY Small And Easy 6	X	72. Loop next to a Multiloop	X
23. Shortie 4	X	73. Snowflake 4	
24. Shape Test	X	74. Mat—Cuboid	
25. The Minitsry	X	75. Misfolded Aptamer 6	X
26. stickshift	X	76. Snowflake 3	
27. U	X	77. Hard Y and a bit more	
28. Still Life (Sunflower In A Vase)	X	78. Mat—Lot 2–2 B	
29. Quasispecies 2–2 Loop Challenge	X	79. Shapes and Energy	
30. Corner bulge training	X	80. Spiral of 5's	
31. Spiral	X	81. Campfire	
32. InfoRNA bulge test	X	82. Anemone	X
33. Worm 1		83. Fractal 3	
34. just down to 1 bulge	X	84. Kyurem 5	X
35. Iron Cross		85. Snowflake Necklace (or v2.0)	
36. loops and stems	X	86. Methaqualone C16H14N2O Structural Representation	
37. Water Strider	X	87. Cat's Toy 2	
38. The Turtle(s) Move(s)		88. Zigzag Semicircle	
39. Adenine	X	89. Short String 4	
40. Tripod5	X	90. Gladius	
41. Shortie 6	X	91. Thunderbolt	
42. Runner	X	92. Mutated chicken feet	
43. Recoil	X	93. Chicken Tracks	X
44. [CloudBeta] An Arm and a Leg 1.0	X	94. Looking Back Again	
45. [CloudBeta] 5 Adjacent Stack Multi-Branch Loop	X	95. Multilooping 6	X
46. Triple Y	X	96. Cesspool	
47. Misfolded Aptamer	X	97. Hoglafractal	
48. Flower power	X	98. Bullseye	
49. Kudzu	X	99. Shooting Star	
50. "1,2,3and4bulges"		100. Teslagon	

We observed that nearly all of the puzzles fell into two categories: ones the algorithm solves quickly and easily, and ones that it simply is not capable of solving (or that require much longer than 24 hours to solve). Of the 60 puzzles it successfully solved, the majority (38) required less than one minute, and nearly all (56) required less than three hours. Running for the full 24 hours yielded only two additional solutions. Running another four tests for each of the remaining puzzles yielded solutions to only two more. This suggests it is well past the point of diminishing returns, and simply running the algorithm longer, or speeding up the computation by any small constant factor, would be unlikely to significantly improve the success rate.

**Fig 2:**
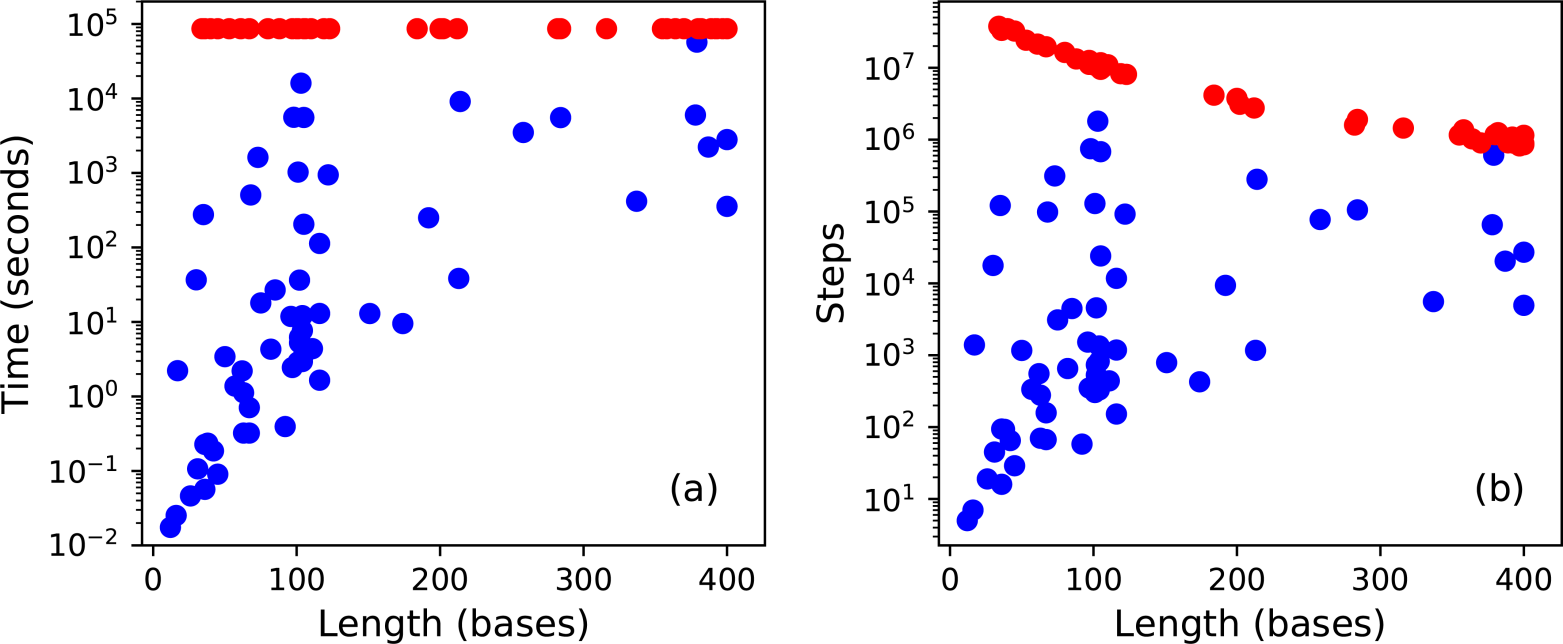
Difficulty of solving puzzles as measured by (a) clock time or (b) steps, versus sequence length. Blue dots represent the 58 puzzles that were successfully solved on the first attempt. Red dots represent the 42 puzzles on which it gave up after running for 24 hours. (Two more of them were eventually solved on later attempts.) Longer sequences take more time per step, so fewer steps can be completed in 24 hours.

All else being equal, one would expect the difficulty and required computation time to increase with sequence length. Fig 2 shows the relationship between these factors. It is clear that computation time does increase with sequence length, but there also is enormous variation. The numbers of steps required to solve two puzzles of the same length may differ by many orders of magnitude. Our method successfully solves some of the very longest puzzles, yet fails to solve some very short ones. In total, it solves 71% (47 of 66) of the puzzles shorter than 150 bases, and 38% (13 of 34) of the ones longer than 150 bases. This suggests sequence length is one factor affecting difficulty, but other factors are equally or more important.

On the other hand, the method's performance on each puzzle very closely matches how difficult that puzzle is for human players. The puzzles are ordered by the number of human players who successfully solved each one. Our method solves every one of the first 32 puzzles, and 46 of the first 50, but only 14 of the final 50. That is, it easily solves all the puzzles that are easy for expert human players, but fails to solve most of the ones that are difficult for expert humans. This suggests the intriguing possibility that the strategies learned by the policy network may be very similar to the strategies followed by human players.

Anderson-Lee et al. identified a set of structural elements that make puzzles difficult to solve [18]. We now consider how well our method handles each of those elements.

### Short stems

**Fig 3:**
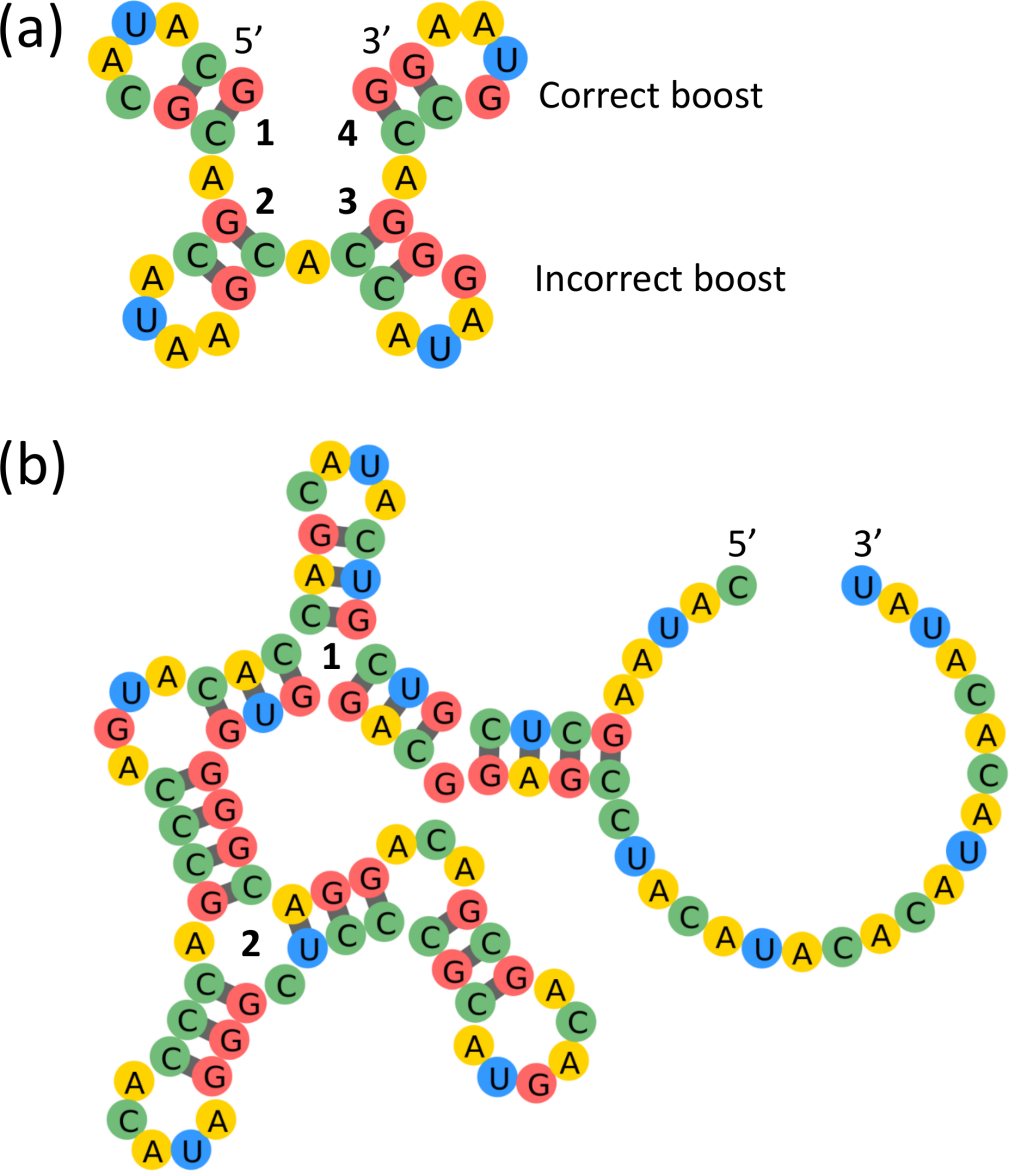
Examples of puzzles with short stems solved by our model: Shortie 6 (a), and Kyurem 7 (b). The Shortie 6 solution strategy involved introducing asymmetric base pairing patterns of GC/CG for stems 1 and 2 and CG/CG for stems 3 and 4. Upon first glance, it seems that the model has successfully learned how to boost 4-loops, since it makes G-mutations at edge bases of the 4-loops at stems 3 and 4. However, while stem 4 is boosted correctly, the G-mutation in the 4-loop of stem 3 is actually in the wrong position and does not stabilize the loop. Kyurem 7 is significantly more difficult due to the presence of two multiloops joining the short stems, but our model successfully proposes asymmetric stem designs around each multiloop to stabilize the structure. Multiloop 1 consists of three stems with a common pattern of alternating GC/AU pairs, whereas multiloop 2 consists of stems composed of almost entirely GC pairs, except for the single AU closing pair in the third stem. Mutations to all but four base pairs in Kyurem 7, highlighted in red, result in misfolding of the structure, indicating the need for precise design of the stem base pairings.

Structures with short stems are in general challenging for design due to their energetic instability [18]. Furthermore, as the number of stems increases, the design difficulty also increases due to the higher probability of mispairing between stems [18]. We observe that our model is competent at addressing these structural challenges. For example, our model can quickly solve both Shortie 4 and Shortie 6 from the Eterna100, which consist of two and four length-2 stems respectively. Shortie 4 took 1390 steps and 2.2 seconds to solve, whereas Shortie 6 required 121,113 steps and a little more than 4 minutes. This is consistent with the fact that Shortie 6, having a larger number of stems, is more difficult to solve. To address the issue of mispairing between stems, our model introduces asymmetries in the stem base pairing patterns, such that stems 1 and 2 are composed of alternating GC/CG pairs, whereas stems 3 and 4 contain non-alternating CG/CG pairs, as shown in Fig 3. The application of different sequences to symmetric structural elements is also a common strategy employed by human players to solve difficult puzzles with high symmetry [18]. Furthermore, our model also chose to mutate the first base at the 5' end of the 4-loop of stem 4 to G, which corresponds to a human-developed strategy that stabilizes stems, named "boosting" by Eterna players. From this observation, we initially believed that our model had learned how to boost 4-loops during training. However, this is not the case, as the model also makes a deceptively boost-like G-mutation at the 3' end of the 4-loop of stem 3, which does not actually stabilize the structure (the correct move would have been to mutate the first base at the 5' end to G). Therefore, we believe that the boost made at stem 4 was not due to the model learning this strategy, but rather due to random chance because of the relatively small size of the puzzle and the large number of steps taken to solve it.

To further investigate whether the model had learned how to boost, we chose 124 structures at random from the validation set that contained 4-loops and examined the distribution of bases at the boosting position. We observed that the boost position has an identity of G in only 19 (15%) of the solutions, suggesting that the model has not learned that boosting is a favorable move. To address the alternative possibility that the model is intentionally not boosting the 4-loop and instead choosing an alternative strategy incompatible with boosting, for all structures in which the boost position was not G, we manually mutated this base to G and then compared the predicted free energy of this boosted sequence to that of the original sequence. We observe that the boost stabilizes the structure an overwhelming majority of the time (98.2% of the structures) by an average of -0.67 ± 0.58 kcal/mol. Therefore, we conclude that the model is not making design choices incompatible with boosting, but rather has not learned the boosting strategy. This is likely because the structures in the training set are small enough that boosting if often not necessary for stabilizing the structure. Indeed, of the 19 predicted solutions with G in the boost position, removing the boost by mutating the G to A did not affect the predicted structure in all but 3 cases, confirming that for the large majority of structures, boosting is not necessary to the solution. In the future, expanding the training set to include longer sequences to make boosting a necessary stabilization move may allow the model to learn this strategy.

In addition to the Shortie puzzles, our model most notably also succeeds in solving both Kyurem 7 and Kyurem 5 very quickly, two structures which none of the algorithms previously benchmarked could solve. These structures are noted for being particularly difficult due to the presence of multiple short stems connected by multiloop junctions, which significantly increases the probability of mispairing between stems. Kyurem 5, the easier of the two puzzles, took our algorithm 821 steps and 7.7 seconds to solve, whereas Kyurem 7 took 4563 steps and 36.4 seconds. Like its solution strategy for Shortie 6, we notice that our model makes asymmetric design choices for Kyurem 7 with respect to the stems surrounding the two multiloops. It assigns the three length-3 stems around the first multiloop a common motif of alternating GC/AU/CG pairs, whereas for the stems around the second multiloop, except for the closing AU pair at (47,67), the model assigns only GC pairs. Previously, it was shown that stabilization of a given stem for Kyurem 7 requires not only making stabilizing mutations within that stem, but also making precise mutations to multiple nearby stems [18]. Thus, such asymmetric design of stems to prevent inter-stem mispairing is likely necessary for stabilizing the structure. We also observe that our proposed sequence is indeed very sensitive to small perturbations. Taking each base pair in the sequence and either swapping the bases or mutating the pair to GC, both common design moves, almost always resulted in the sequence misfolding into a different structure. Only perturbations of four base pairs: (15,20), (36,44), (37,43), or (51,60) did not result in misfolding.

Finally, in a striking contrast with previous computational algorithms, we note that our model can solve both Kyurem 7 and Kyurem 5 much faster than Shortie 6, which is designed to be a less difficult puzzle. This raises the possibility that perhaps our model is not learning design strategies in a progressive manner, i.e. simple to advanced, but is somehow first learning advanced strategies, leaving a gap in its knowledge base that makes it more difficult to solve easier puzzles.

### Bulges and internal loops

The presence of bulges and internal loops are known to destabilize structures, as they result in a smaller overall amount of base pairing and encourage mispairing to form more stable stems [18]. Likewise, our model's performance noticeably decreases as the number of bulges or internal loops increase. For example, our model can solve the relatively easy "Just down to 1 bulge" extremely quickly in only 11.9 seconds, but fails for its more difficult counterpart "1,2,3and4bulges". This is consistent with the performance of previous computational algorithms: "1,2,3and4bulges" was only solvable by MODENA, whereas "Just down to 1 bulge" was solvable by 4 of 6 algorithms. Likewise, our model succeeds in solving "Loop next to a multiloop", which consists of two internal loops stabilized by a short stem, in about 25 minutes, but fails for structures such as "Crop circle 2", which consists of five internal loops stabilized by short stems. This is consistent with the performance of previous computational algorithms, as "Loop next to a multiloop" was solvable by RNA-SSD, but "Crop circle 2" was unsolvable by any algorithm. We expect that structures such as "Crop circle 2", due to their complexity and uniqueness, will pose challenges for our model, as such motifs have not been encountered during training.

### Symmetric structural elements

As discussed above, structures with high symmetry introduce opportunities for mispairing between symmetric elements, contributing to design difficulty. Our model shows varied success at solving puzzles with this characteristic. Although it can readily handle simple puzzles with symmetric elements such as Shortie 6 and Fractal 2, it fails for more complicated puzzles such as Mutated Chicken Feet, which consists of three symmetric branches of consecutive short stems linked by bulges and multiloops, a combination of several factors that make design difficult. The unique combination of different structural elements comprising this puzzle poses a significant challenge to our current model, since it is distinct from anything encountered in the training set.

### Infrequently occurring structural patterns

Unusual structural patterns that arise infrequently in nature also present challenges for current design algorithms. For example, in the "Hard Y" puzzle, the presence of two consecutive small bulges in a structure, named the "zigzag", significantly increases the difficulty of the puzzle and renders it unsolvable for any of the previously benchmarked computational algorithms. Remarkably, we observed that our model could propose a valid sequence to stabilize the zigzag and solve "Hard Y" in a little under 10 minutes. However, given the relatively long timescale taken to solve hard Y compared to puzzles of even significantly longer length such as the Kyurem puzzles, it is possible that our model did not actually learn the solution strategy for the zigzag during training, but instead arrived at the solution primarily by random search after intelligently solving the remainder of the puzzle.

### Overcomplicating the design strategy

Many computational algorithms approach the RNA design problem by first initializing to a random sequence of bases and then performing some form of stochastic search, such as RNAInverse and RNA-SSD. This random initialization can overcomplicate the solution process for certain structures. For example, "This is actually small and easy 6", which is a chain of 400 unpaired bases, was solvable only by NUPACK and DSS-Opt out of the six methods tested. A trivial solution that is easily identifiable by humans is to simply set every position in the sequence to the same base, but many computational algorithms fail to arrive at this solution due to the random initialization of the sequence, leading to many unintended base pairs. We expected our model, which also randomly initializes the sequence, to struggle with this structure. However, our model was able to solve this puzzle remarkably quickly in less than 5000 steps despite the structure's length. This indicates that the model is not making random moves, but has learned during training how to break unwanted base pairs and is making intelligent mutation choices to fix a bad initial sequence.

In summary, we observe that our model is very competent at stabilizing short stems, even in the presence of additional destabilizing structural motifs such as multiloops or structural symmetry, by introducing precise asymmetric base pairing patterns for the stems. The two representative puzzles with these characteristics unsolvable by any previous computational algorithm, Kyurem 5 and Kyurem 7, were both solved very quickly by our model.

On the other hand, our model seems to struggle with structures containing large numbers of bulges or internal loops. Although it solves the relatively simple "Just down to 1 bulge", our model fails for the more difficult "1,2,3,and4bulges" and "Crop circle 2". We hypothesize that the model's poor performance on these structures is because stabilization of them requires the application of strategies the model did not learn during training. For example, "1,2,3,and4bulges" possesses an unusual structural motif of a 4-loop connected to a length-1 stem that to our knowledge must be boosted to stabilize the structure. Likewise, "Crop circle 2" requires the internal loops to be simultaneously boosted in one of few specific ways. Because our model did not learn the boosting strategy during training, it expectedly fails for structures where boosting is necessary for stabilization. As a future study, increasing the length of the structures in the training set may allow for sampling of more complicated structures for which boosting is a necessary stabilization move. This would allow the model to consistently sample positive rewards for boosting during training and learn the strategy.

### Conclusion

In this study, we train an agent for RNA design using reinforcement learning and a training set of randomly generated secondary structures. We observe that the agent can learn effective design strategies fully autonomously with no human input. Remarkably, our trained agent outperforms any previous computational algorithm, solving 60/100 of the Eterna100. Due to the limited length and complexity of our current training set, the model is unable to learn certain stabilizing strategies such as boosting, hindering its performance on more difficult structures for which these strategies are necessary. Expanding the length of the training sequences may allow the model to sample more complex structural motifs and learn more advanced strategies to improve its performance. We plan to investigate this possibility in future work.

## Supporting information

S1 Solutions Solutions to the Eterna100 puzzles found by our method. (CSV)

S1 Source code Source code and data to reproduce the results of this paper. (ZIP)
